# Survivin Mutant Protects Differentiated Dopaminergic SK-N-SH Cells Against Oxidative Stress

**DOI:** 10.1371/journal.pone.0015865

**Published:** 2011-01-10

**Authors:** Sara Baratchi, Rupinder K. Kanwar, Jagat R. Kanwar

**Affiliations:** Laboratory of Immunology and Molecular Biomedical Research, Centre for Biotechnology and Interdisciplinary Biosciences, Institute for Technology Research and Innovation, Deakin University, Waurn Ponds, Victoria, Australia; Boston University School of Medicine, United States of America

## Abstract

Oxidative stress is due to an imbalance of antioxidant/pro-oxidant homeostasis and is associated with the progression of several neurological diseases, including Parkinson's and Alzheimer's disease and amyotrophic lateral sclerosis. Furthermore, oxidative stress is responsible for the neuronal loss and dysfunction associated with disease pathogenesis. Survivin is a member of the inhibitors of the apoptosis (IAP) family of proteins, but its neuroprotective effects have not been studied. Here, we demonstrate that SurR9-C84A, a survivin mutant, has neuroprotective effects against H_2_O_2_-induced neurotoxicity. Our results show that H_2_O_2_ toxicity is associated with an increase in cell death, mitochondrial membrane depolarisation, and the expression of cyclin D1 and caspases 9 and 3. In addition, pre-treatment with SurR9-C84A reduces cell death by decreasing both the level of mitochondrial depolarisation and the expression of cyclin D1 and caspases 9 and 3. We further show that SurR9-C84A increases the antioxidant activity of GSH-peroxidase and catalase, and effectively counteracts oxidant activity following exposure to H_2_O_2_. These results suggest for the first time that SurR9-C84A is a promising treatment to protect neuronal cells against H_2_O_2_-induced neurotoxicity.

## Introduction

Parkinson's disease (PD) is a chronic and progressive neurodegenerative disorder, in which dopaminergic (DArgic) neurons in the substantia nigra are selectively degenerated. This degeneration leads to the formation of fibrillar cytoplasmic inclusions known as Lewy bodies (LBs) [1]. Oxidative stress is a critical factor in this disease, as shown by different studies including direct analysis of postmortem samples and indirect demonstrations of oxidative stress capacity in inducing nigral cell loss [2], [3]. Oxidative stress results from insufficient scavenging of reactive oxygen species and is reported to be the cause of the selective degeneration of DArgic neurons in PD through both mitochondrial dysfunction and apoptosis [4]. Oxidative stress occurs in DArgic neurons due to the metabolism of dopamine, which generates various molecules such as hydrogen peroxide, superoxide radicals and dopamine-quinone that act as endogenous toxins [5].

Although the exact mechanism underlying the degeneration of DArgic neurons in PD is not currently clear, mitochondrial dysfunction, genetic mutations, protein aggregation, and ultimately apoptosis are the major contributing factors that have been identified so far [6].

There is an increasing interest in using inhibitors of apoptosis (IAP) family proteins to target different aspects of degenerative diseases. Reportedly, adenoviral delivery of NAIP, HIAP1 and HIAP2 has shown protective effects on ischemic damage [7] and sciatic axotomy [8]. Moreover, the BH4 domain of Bcl-x attached to TAT, a membrane transport peptide, has a neuroprotective effect against acute hypoxia/ischemia injury [9]. Using wild type IAP family proteins in human trials always raises concerns due to their role in cancer formation [10], [11], [12] and in the induction of mitosis in postmitotic neurons. Alternatively, developing IAP mutants capable of protecting neurons will provide insight into the treatment of degenerative diseases of the brain.

Survivin is a unique member of the IAP family and has an intriguing function in the chromosomal passenger complex (CPC). It contributes to microtubule instability and is necessary for both the correct alignment of chromosomes on mitotic spindles and biorientation (the capture of sister kinetochores by microtubules from opposite spindle poles) prior to anaphase [13]. Survivin has a dominant role in the inhibition of apoptosis through dimerisation with its co-factors XIAP and hepatitis B X-interacting protein (HBXIP) [14], [15]. Although these unique features make survivin an ideal target for neuroprotection and proliferation, no efforts have been made to study its subcellular network during neurodegenerative diseases and its potential use as a target for neuroprotection. Previously we found the SurR9-C84A has neuroprotective effect against the post differentiation retinoic acid induce cell death and cytotixic effect of activated T-cells supernatant [16], [17].

In the present study, we demonstrate that pre-treatment with SurR9-C84A can protect the differentiated DArgic such as neuroblastoma SK-N-SH cells against H_2_O_2_-induced oxidative damage in terms of intracellular redox and cellular death. Here, we demonstrate that pre-treatment with SurR9-C84A can protect differentiated DArgic cells such as neuroblastoma SK-N-SH cells against H_2_O_2_-induced oxidative damage in terms of intracellular redox and cell death. We also report the ability of survivin to activate antioxidant scavengers, including GSH-peroxidase (GSHPx), GSH-reductase (GSHR), GSH-transferase (GST), superoxide dismutase (SOD), and catalase (CAT).

## Materials and Methods

### Cell line and culture conditions

Human SK-N-SH, obtained from the American Type Culture Collection (ATCC) were grown as a monolayer in the Dulbecco's Minimum Essential Medium (DMEM) media supplemented with 10% of heat-inactivated Foetal Bovine Serum (FBS), penicillin (20 units/ml) and streptomycin (20 mg/ml) at 37°C in a saturated humid atmosphere with 5% CO_2_. As the cells became confluent, they were split after treatment with Trypsin-EDTA.

To determine cell viability and mitochondrial depolarization SK-N-SH cells were differentiated in 96 well plate at 10^4^ cells per well. For TUNEL assay SK-N-SH cells were seeded in BD Falcon™ 8-well culture slides (surface area 0.7 cm^2^/well) at 10^4^ cells/well. To evaluate GSH enzyme activities cells were seeded in 6 well plates (surface area 2.2 cm^2^/well). To initiate the differentiation in SK-N-SH, cells were grown in the DMEM media containing the 20 µM retinoic acid (RA) (Sigma-Aldrich) under the dark conditions with replacement of the conditioned media every 48–72 hr for two weeks. Cells were considered to be differentiated if they had at least one process longer than the cell body regarded as neurite [18].

Before any treatment, the differentiation media were replaced with DMEM media. All experiments were performed after 24 hr of incubation at 37°C in 5% CO_2_. In order to evaluate the effect of SurR9-C84A on viability, differentiated SK-N-SH cells were treated with different concentration of SurR9-C84A (10–100 µg/ml) for 24 hr. Similarly, cells were treated with different concentration of H_2_O_2_ (10–500 µM) for 24 hr. The 75 µg/ml concentration of SurR9-C84A had the best effect on viability and 300 µM concentration of H_2_O_2_ had the significant cytotoxic effect, and therefore were selected to study the neuroprotective effect of SurR9-C84A. All the SurR9-C84A incubation was 24 hr a head of H_2_O_2_ therapy. As a control, ascorbic acid was used with the same treatment condition of SurR9-C84A therapy.

### Construction of BIR motif mutant of Survivin expressing vector and protein purification

Construction, purification and internalization of dominant-negative cell-permeable (9 arginine residues) form of survivin mutant (SurR9-C84A) have been fully explained in our previous works [19], [20].

### Cell viability assay

To evaluate the level of cell toxicity, MTT assay was conducted. The colorimetric MTT (3-(4, 5-dimethylthiazol-2yl)-2, 5-diphenyltetrazoliumbromide) assay was conducted with differentiated SK-N-SH cells. Differentiated cells were pre-treated with/without 75 µg/ml of SurR9-C84A or ascorbic acid for 24 hr followed by treatment with 300 µM H_2_O_2_ for 24 hr prior to MTT assay. Each experiment was conducted in triplicate wells. Mean (±SEM) values were calculated from at least three independent experiments.

### Lactate Dehydrogenase (LDH) Release Assay

Apoptotic and necrotic cells release LDH in the media. Difference in the LDH concentration was measured using cytotoxicity detection kit (Roche). Briefly, differentiated cells were pre-treated for 24 hr with 50 µg/ml SurR9-C84A, 50 µg/ml ascorbic acid and then treated with 300 µM H_2_O_2_ for 3 hr. For analysis, 100 µl supernatant was extracted from each well and placed in separate 96-well plate. The 100 µl catalyst solution was added to each well and incubated at 37°C for 30 min. Absorbance was measured at 490 nm using a microplate reader. Total cellular LDH was determined using total cell lysate following 2% triton X-100. The assay medium served as a low control and its absorbance was subtracted from all absorbance measurement to estimate the cytotoxicity (%) as: 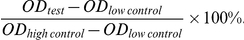


### Western blotting

For western blot analysis, 70 µg of differentiated cell lysate protein was used. Cell lysate was prepared using the RIPA buffer (150 mM sodium chloride, 1.0% NP-40, 0.5% sodium deoxycholate, 0.1% SDS and 50 mM Tris, PH 8.0) containing protease inhibitor tablets (Roche Applied Science). The total protein content was measured using the Bradford assay. Samples were electrophoresed and transferred to the PVDF membrane. Primary antibodies used were goat anti-β-actin (1/1000, Santa Cruz) and mouse anti-cyclin D1 (1/2000, Cell signalling). Secondary antibodies were peroxidase conjugate anti-mouse (1/2000, Cell signalling), peroxidase conjugate anti-goat (1/10000, sigma Aldrich). The blots were developed using ECL system (Amersham Biosciences, Arlington Heights, IL, USA).

### Determination of caspase 9 and 3 activities

Caspase enzyme activity was determined as reported previously. Briefly treated differentiated cells were washed with PBS and cell lysate was prepared using the lysis buffer (150 mM sodium chloride, 1 mM ethylene diamine tetraacetate (EDTA), 1 mM ethylene glycol-bis(2-aminoethyl)-N,N,N',N'-tetraacetic acid (EGTA), 1% Triton X-100) containing protease inhibitor tablets (Roche Applied Science). Caspase activity was measured using the cell signaling PathScan® Sandwich ELISA techniques following the supplier instruction. The resulting absorbance was read at 490 nm on an ELISA plate reader (Ansys, Expert plus).

### Determination of the intrinsic Mitochondrial Membrane Potential (Δ**ψm)

Mitochondrial membrane potential variation was determined using the MitoLight Mitochondrial Apoptosis Detection Kit (Chemicon) according to the manufacturer's instruction. MitoLight is a lipophilic cationic dye, which stains living cell mitochondria according to their membrane potentials. In healthy cells, the dye accumulates and aggregates in the mitochondria and fluoresces red (Em = 585−590 nm) while in apoptotic cells, the dye remains in the cytosol and fluorescence green (Em = 527−530 nm).

Briefly, cells were washed with PBS. Diluted mitolight reagent was added to the cells and incubated at 37°C in 5% CO_2_ for 20 min. Cells were washed with PBS and the fluorescent were measured at λ_excitation_ = 485 nm and λ_emission_ = 580 nm with a spectrofluorometer. The ratio of red (Em = 585–590 nm) to green (Em = 527–530 nm) reflects the Δψm.

### TUNEL staining

TUNEL (terminal deoxynucleotidyl transferasemediated dUTP-biotin nick end-labeling) staining performed using In Situ Cell Death Detection Kit (Roche) following the maufacturer's instruction. Slides were mounted with mounting media (Vector Labs, USA).

### Preparation of cell lysate for determination of enzyme activity

Differentiated SK-N-SH cells were washed three times with cold PBS. Cells were subsequently scraped and collected in 1 mL of PBS. The pallet was lysed by sonication and centrifuged for 15 min at 10000 g at 4°C. The supernatant were used for the determination of enzyme activity analyses after protein concentration determination.

### Determination of enzyme activities

GST activity was determined using the Glutathione S- Transferase Assay Kit (Cayman) according to the manufacturer's instruction. Briefly, GST activity was spectrophotometrically assessed by measuring the conjugation of 1-chloro-2, 4-dinitrobenzene (CDNB) with glutathione at 340 nm. GSHR activity was assessed using the Glutathione Reductase Assay Kit (Cayman) following the manufacturer's instruction and expressed as nmol of NADPH oxidation per min/mg of total cellular protein per assay.

GSHPx, SOD and CAT activities were determined using the GSHPx, SOD and CAT determination Kits (Cayman), respectively, according to the manufacturer's instructions. GSHPx and CAT were defined as the amount of enzyme causes the 1.0 nmol NADPH oxidation and the amount of enzyme causes the formation of 1.0 nmol formaldehyde per min/mg of total cellular protein per assay respectively. SOD activity was calculated using the SOD standard curve and expressed as U/mg of total cellular protein.

### Image and data analysis on cell cultures

Analysis and photography of fluorescent immunostaind cells were carried out using an inverted Leica SP5 confocal microscope. Results are shown as the mean ± standard error of the mean (SEM) of data extracted from three experiments unless stated otherwise. Statistical differences were determined with a one-way analysis of variance (ANOVA) followed by Dunnett's test. *P* values ≤0.01 will be considered significant.

## Results

### SurR9-C84A protects against H2O2 induced cytotoxicity

**Figure 1:**
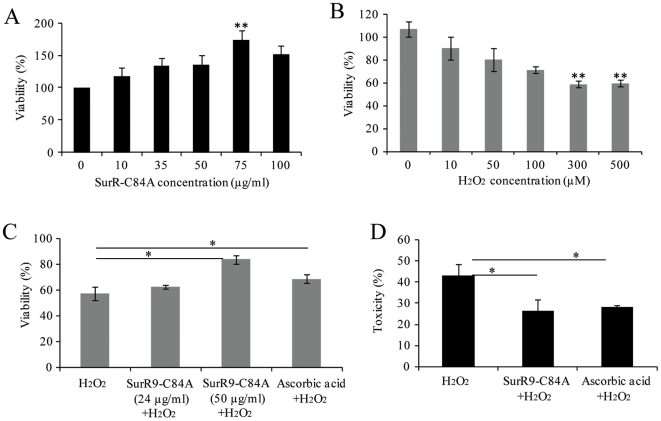
SurR9-C84A attenuate H_2_O_2_ induced cell death. SK-N-SH cells were differentiated using 20 µM retinoic acid for 10 days and ddifferentiated media were replaced with growth media. (A) Differentiated cells were treated with different concentration of SurR9-C84A for 24 hr and cell viability was determined using MTT assay. (B) Differentiated SK-N-SH cells were treated with different concentration of H_2_O_2_ and cell viability was determined using MTT assay. (C, D) Differentiated SK-N-SH cells were pre-treated with 75 µg/ml of SurR9-C84A or ascorbic acid for 24 hr followed by treatment with 300 µM of H_2_O_2_ for 24 hr. The cell viability and toxicity were determined using (C) MTT and (D) LDH assays, respectively. Data are representative of at least three independent experiments and expressed as mean±SEM; *P<0.05, **P<0.01.

Preliminary, to find the effect of SurR9-C84A therapy the differentiated SK-N-SH cells were treated with different concentrations of SurR9-C84A for 24 hr. A dose-dependent increase in the viability of differentiated SK-N-SH cells was found (Fig. 1A).

To evaluate whether H_2_O_2_ affects the viability of differentiated SK-N-SH cells, these cells were treated with different concentrations of H_2_O_2_ (0, 10, 30, 100, 200 and 500 µM) for 24 hr. The H_2_O_2_ treatment led to a dose-dependent decrease in the viability of differentiated SK-N-SH cells (Fig. 1B).

Furthermore, to determine the protective effects of SurR9-C84A, differentiated SK-N-SH cells were pre-treated with 75 µg of SurR9-C84A, followed by treatment with 300 µM H_2_O_2_ for 24 hr. The H_2_O_2_-induced loss of cell viability was significantly attenuated by SurR9-C84A treatment (P<0.05) (Fig. 1C).

To further investigate the protective effects of SurR9-C84A, LDH release was evaluated as a second indicator of toxicity. There was a considerable increase in LDH release following exposure to 300 µM H_2_O_2_ (50±3.4%, P<0.05) (Fig. 1d), indicating an increase in cell toxicity following the H_2_O_2_ exposure. By contrast, pre-treatment with SurR9-C84A for a period of 24 hr significantly reduced LDH release by (30±4.63%, P<0.05). We also compared the protective effect of SurR9-C84A with ascorbic acid in the same experimental condition and found that pre-treatment with ascorbic acid reduces the LDH release by (28±3.42%, P<0.05).

### SurR9-C84A pre-treatment attenuates cell death

**Figure 2:**
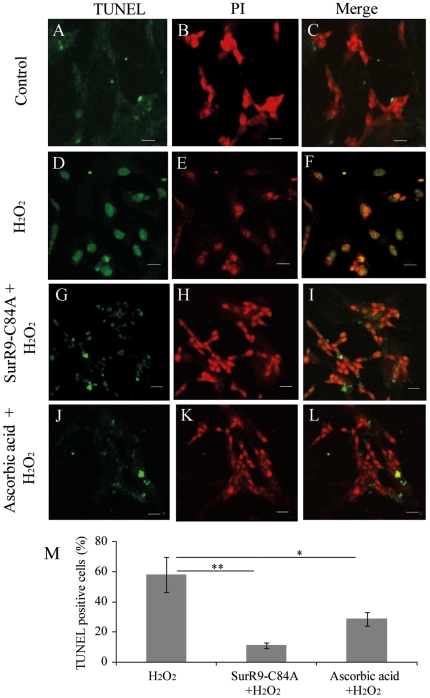
SurR9-C84A prevents H_2_O_2_ induced apoptosis and necrosis in differentiated SK-N-SH cells. SK-N-SH cells were differentiated with 20 µM RA for 10 days. Differentiation media were replaced with growth media and cells were treated with (A–C) 300 µM H_2_O_2_, (D–E) Control, (G–I) 75 µg/ml SurR9-C84A +300 µM H_2_O_2_ and (J–L) 75 µg/ml ascorbic acid +300 µM H_2_O_2_ for 24 hr. (M) graph shows the percentage of apoptotic and necrotic cells. Neuronal cells were stained with TUNEL/Propidium iodine double staining and analysed using confocal microscopy as described in materials and methods. The values are presented as the percentage of total number of cells and shown as mean±SEM of three independent experiments. At least 100 cells were counted in each treatment. Bar is 10 µm.

Considering the high level of apoptotic neuronal loss during PD, we studied the protective effects of SurR9-C84A against H_2_O_2_-induced apoptosis using TUNEL and PI staining assays. Our results showed that oxidative stress increases the population of apoptotic cells to (58±1.69%, P<0.01) compared to the control cells. Pre-treatment with SurR9-C84A significantly reduced the percentage of apoptotic cells by (40±12%, P<0.01) compared to the cells treated with H_2_O_2_ alone whereas pre-treatment with ascorbic acid improved the population of apoptotic cells by (18±12%, P<0.05). These results revealed that SurR9-C84A protects differentiated SK-N-SH cells from H_2_O_2_-induced DNA fragmentation and cell death, highlighting its anti-apoptotic effect on differentiated SK-N-SH cells (Fig. 2).

### SurR9-C84A protects against H_2_O_2_-induced reduction of the mitochondrial membrane potential

To clarify the mechanism underlying the anti-apoptotic effects of SurR9-C84A, we evaluated any reduction in the mitochondrial transmembrane potential as an early apoptotic event, and any increased in cleavage of caspases 9 and 3 and expression of cyclin D1, all markers of neuronal cell death.

**Figure 3:**
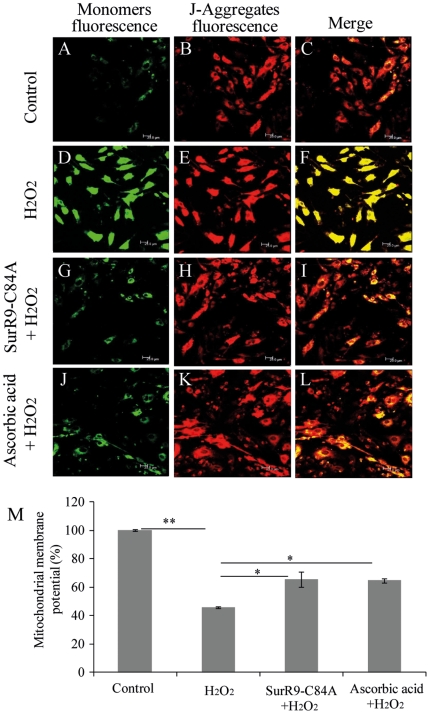
SurR9-C84A prevents mitochondrial depolarization. SK-N-SH cells were differentiated with 20 µM retinoic acid for 10 days. Differentiated media were replaced with growth media and cells were pre-treated with 75 µg/ml of SurR9-C84A or ascorbic acid for 24 hr followed by treatment with 300 µM of H_2_O_2_ for 24 hr. At the end of incubation mitochondrial membrane depolarization was qualified and quantified with MitoLight Mitochondrial kit using both techniques of (A) confocal microscopy and (B) spectrofluorometery (see material and method). Green fluorescence (detection of monomers) indicates the presence of depolarized mitochondria (apoptotic cells). Red fluorescence (J-aggregates) indicates the functional and polarized mitochondria. Values are presented as a percentage of increase in mitochondrial depolarization. Data are representative of at least three independent experiments and expressed as mean±SEM; *P<0.05, **P<0.01.

We evaluated the mitochondrial membrane potential to determine whether H_2_O_2_-induced apoptosis and the protective effects of SurR9-C84A occur through the mitochondrial pathway. Exposing differentiated SK-N-SH cells to H_2_O_2_ decreased the mitochondrial membrane potential to 54.13±4.32% (p<0.01) (Fig. 3D–F, M). Pre-treatment with SurR9-C84A for 24 hr, however, increased the mitochondrial membrane potential to 65±6.29% (p<0.05) (Fig. 3G–I, M) compared to the cells treated with H_2_O_2_ alone whereas pre-treatment with ascorbic acid improved the mitochondrial membrane potential to 64.6±8.9% (p<0.05) (Fig. 3J–L, M). These results showed that the pre-treatment of differentiated SK-N-SH cells with SurR9-C84A decreases the H_2_O_2_-induced mitochondrial membrane potential, which was comparable to results obtained by ascorbic acid.

**Figure 4:**
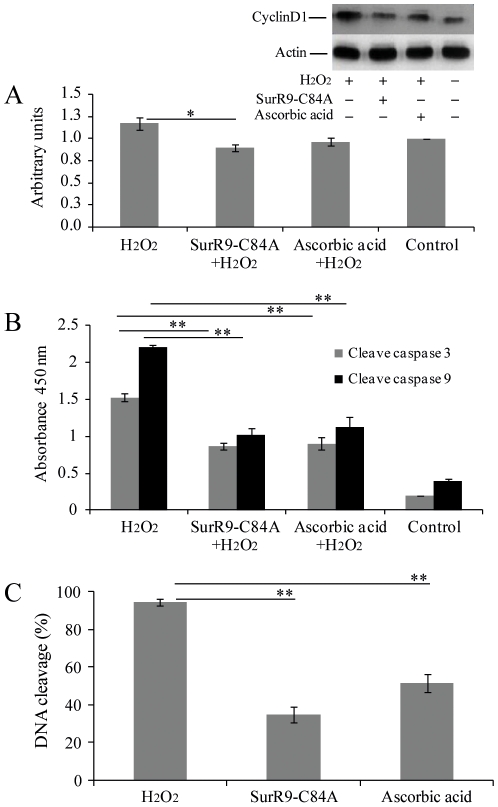
SurR9-C84A pre-treatment prevents expression of neuronal cell death markers such as cyclin D1, caspase 9 and 3. SK-N-SH cells were differentiated for 10 days with 20 µM RA. After the differentiation period the media was replaced with cell growth media and cells were pre-treated with 75 µg/ml SurR9-C84A or 75 µg/ml ascorbic acid for 24 hr followed by treatment with 300 µM H_2_O_2_ for 24 hr. (A) cell lysate was prepared and western blot analysis was performed to study the expression of cyclin D1. The loading of each lane was normalised to the level of β-Actin. (B) ELISA assay was performed for cleavage of caspase9 and 3 as described in materials and methods. SurR9-C84A protects the nucleus damage. Cells were pre-treated with 75 µg/ml SurR9-C84A or ascorbic acid for 24 hr followed by treatment with 300 µM H_2_O_2_ for 24 hr and stained with PI. Arrows show the damage nuclei. (C) Percentage of cells with abnormal nuclei.

In addition, using the same experimental conditions, pre-treatment with SurR9-C84A showed an inhibitory effect on the activation of caspases 9 and 3. As shown in Fig. 4, exposure of cells to the H_2_O_2_ did not affect the expression of cyclin D1 while significantly increased cleavage of caspase 9 (6±0.30 fold; P<0.01) and caspase 3 (7±0.20 fold; P<0.01). Furthermore, pre-treatment with ascorbic acid reduced the activation of caspase 9 (51±13.6%; P<0.01) and caspase 3 (60±8.6%; P<0.01) while no significant difference was observed in the expression of cyclin D1.

More importantly, pre-treatment with SurR9-C84A showed a significant inhibitory effect on the cleavage of caspase 9 (55%±5.36; P<0.01) and caspase 3 (35%±0.89; P<0.01) and also on the expression of cyclin D1 (52%±6.4; P<0.05).

With regard to the late stage of apoptosis, we evaluated the inhibitory effect of SurR9-C84A on DNA fragmentation. A 24 hr treatment with 300 µM H_2_O_2_ increased the DNA fragmentation to 88.3±7.26% (P<0.05). Interestingly, pre-treatment with SurR9-C84A for 24 hr reduced the DNA fragmentation to 34.5±5.4% (p<0.01). Alternatively, pre-treatment with ascorbic acid reduced the DNA fragmentation to 56.7±6.32 (P<0.01), which was 25% less effective compared to the SurR9-C84A pre-treatment.

### SurR9-C84A improves antioxidant activity of differentiated SK-N-SH cells

To determine whether the observed neuroprotective effect of SurR9-C84A could be translated to antioxidant activity, we studied the ability of SurR9-C84A to induce GSH related enzymes. As shown in Table 1, pre-treatment with 75 µg/ml of SurR9-C84A significantly increased the activity of GSHR (2.37±0.06 fold, P<0.05), GST (1.46±1.11 fold, P<0.05) and GSHPx (1.25±0.31 fold, P<0.05) compared to the control level. We next considered the activities of other enzymes, including SOD and CAT, which are involved in the specific detoxification of H_2_O_2_. Notably, a significant increase in the activity of SOD was observed following treatment with SurR9-C84A (1.53±0.005 fold, P<0.05).

**Table 1:** Effects of SurR9-C84A treatment on SOD, CAT, GST, GSHPx and GSHR activity on differentiated SK-N-SH cells.

	Control	SurR9-C84A	H_2_O_2_	SurR9-C84A +H_2_O_2_
SOD (U/mg)	0.032±0.005	0.049±0.002*	0.61±0.03	0.52±0.02
CAT(nmol/min/mg)	0.84±0.05	1.16±0.04	0.21±0.03	0.91±0.05**
GST (nmol/min/mg)	13.09±0.01	19.12±1.1*	9.94±0.2	11.89±0.3*
GSHPx (nmol/min/mg)	1.06±0.02	1.32±0.11*	0.58±0.07	0.69±0.03
GSHR (nmol/min/mg)	2.58±0.05	6.12±0.01**	1.52±0.02	4.35±0.01**

In order to ascertain whether the increase in antioxidant activity following SurR9-C84A treatment could be translated into cytoprotective effects, we studied the protective effects of SurR9-C84A pre-treatment against H_2_O_2_ toxicity. During the oxidative stress condition the level of enzyme activity reduced in GSHR (33±1.56%, P<0.01), GSHT (29.48±4.69%, P<0.05) and GSHPx (40.64±4.69%, P<0.01) and the reduction was replenished corresponding to SurR9-C84A therapy. Pre-treatment of differentiated SK-N-SH cells with 70 µg/ml SurR9-C84A for 24 hr had a significant protective effect against H_2_O_2_ toxicity, and this result was considerably correlated with the increased activity of GSHR (2.8±0.03 fold, P<0.01) and GSHT (1.19±0.5 fold, P<0.05) compared to the H_2_O_2_ treated cells.

## Discussion

Exit from the cell cycle is the most important feature of differentiated neurons that makes them different from the other cells [21]. Consequently, kinases, transcription factors, signalling molecules and regulators of the cell cycle that are active in the other cells seem to be inactivate in routine neuronal biochemistry. Failure to exit the cell cycle and activation of a molecular cell cycle program by DArgic neurons has been reported in both PD and AD. For example, cyclin D1 expression has been observed in DArgic neurons in the postmortem substantia nigra pars compacta (SNc) of PD patients [22], [23], [24], [25], [26]. In addition, different genes underlying the familial forms of PD have been linked to cancer and cell cycle regulation [23]. Therefore, targeting cell cycle markers seems to be a promising approach to protect the SNc from oxidative damage [21].

We report here for the first time a neuroprotective effect of a cell-permeable form of a survivin mutant (SurR9-C84A) against H_2_O_2_-induced neurotoxicity. The cysteine at position 84 in the zinc-coordination site of SurR9-C84A was replaced with alanine, and the amino-terminus was fused to a nine-arginine (R9) peptide to render it cell permeable [19]. Using the R9 peptide has advantages over other carriers due to its greater cellular uptake [27]. It is also superior to other viral systems because it is less toxic and does not induce a significant immune response [28], an important point for future *in vivo* studies.

SurR9-C84A was employed to investigate the survival mechanisms in differentiated DArgic such as neuroblastoma SK-N-SH cells following the H_2_O_2_-induced apoptosis, which has not been studied previously.

The SurR9-C84A protein has been shown to bind to polymerised microtubules and to localise to the microtubule organising centre of interphase cells in a manner indistinguishable from that of wild-type survivin [29]. We previously reported that SurR9-C84A has a protective effect against post differentiation RA-induced cell death [20]; however, no study has been done on neuroprotective therapy with this mutant against oxidative injuries. Hydrogen peroxide is a central molecule involved in the neuronal loss observed in both AD and PD [30]. In this regard, our results show that pre-treatment with SurR9-C84A can increase the viability of neuronal cells and inhibit cell death due to H_2_O_2_-induced damage. Based on previous results, deregulation of cell cycle markers can elicit apoptosis in post mitotic neurons, which is one of the primary mechanisms leading to cell death in degenerating neurons, including DArgic neurons [23]. We also found an increase in cyclin D1 expression following H_2_O_2_ treatment; however, pre-treatment with SurR9-C84A blocked this increase.

Based on previous *in vitro* and clinical studies beta amyloid (Aβ), dopamine and 6-OHDA can lead to cell death by direct H_2_O_2_-induced mitochondrial transmembrane depolarisation and the subsequent activation of caspases 9 and 3 [30]. In the present study, treatment of differentiated SK-N-SH cells with H_2_O_2_ also resulted in a significant increase in the population of apoptotic and necrotic cells, mitochondrial membrane depolarisation and activation of caspases 9 and 3. More importantly, we demonstrated that pre-treatment of differentiated SK-N-SH with SurR9-C84A induces marked resistance to neuronal death in terms of the apoptosis and necrosis elicited by H_2_O_2_. In particular, we showed for the first time that the neuroprotective effects of SurR9-C84A can be attributed to its ability to prevent mitochondrial depolarisation and the activation of apoptotic signalling mediated by caspases 9 and 3, which are responsible for DNA damage. Several different strategies have been proposed to limit oxidative stress, including ascorbic acid therapy. As a control, we compared the effectiveness of our treatment to ascorbic acid and found a superior protective effect of SurR9-C84A against apoptosis and necrosis, mitochondrial membrane depolarisation and activation of caspases 9 and 3.

The involvement of oxidative stress in Parkinson's and Alzheimer's disease and amyotrophic lateral sclerosis has been extensively suggested in the literature. For example, GST has been reported to be involved in the survival of DArgic neurons in PD [31], [32], whereas CAT and SOD have been reported to have important roles in cellular defence against oxidative stress as decreases in their activity have been observed in parkinsonian brains. To evaluate the effects of our treatment on GST, GSHR, GSHPx, CAT and SOD, we studied the activation of these enzymes following SurR9-C84A treatment and found an increase in GST, GSHR and CAT activity, but not SOD activity.

Given that this is the first report of a protective effect of SurR9-C84A following an oxidative stress injury, further work should be done to study the effects of SurR9-C84A on *in vivo* models of degenerative diseases. In summary, we report for the first time, a recombinant, cell-permeable form of the survivin mutant protein (SurR9-C84A) efficiently enters neuronal cells, protects differentiated SK-N-SH cells from the activation of apoptosis induced by H_2_O_2_, decreases the expression of cell cycle markers, and increases antioxidant activity. Emerging nano-delivery systems could be used to bypass the blood brain barrier, facilitating drug delivery to the damaged brain [33].
