# Correction: Survivin Mutant Protects Differentiated Dopaminergic SK-N-SH Cells Against Oxidative Stress

**DOI:** 10.1371/annotation/c6435c2d-0692-476f-8795-d8e49421cd3c

**Published:** 2011-02-01

**Authors:** Sara Baratchi, Rupinder K. Kanwar, Jagat R. Kanwar

There was an error in the Author Contributions. The correct Author Contributions are: Designed the experiments: JRK RKK SB. Performed the experiments: SB. Analyzed the data: JRK RKK SB. Contributed reagents/materials/analysis tools: SB RKK JRK. Wrote the paper: JRK RKK SB. This research work is the part of PhD thesis (SB) under the supervision of JRK RKK.

