# Correction: Survivin Mutant Protects Differentiated Dopaminergic SK-N-SH Cells Against Oxidative Stress

**DOI:** 10.1371/journal.pone.0194587

**Published:** 2018-03-15

**Authors:** Sara Baratchi, Rupinder K. Kanwar, Jagat R. Kanwar

The authors would like to correct Fig 3. In Fig 3, the panels used for 3A-C are incorrectly taken from the same micrograph as 3G-I. The authors have provided a corrected version of Fig 3 here. The authors confirm that these changes do not alter their findings.

**Fig 3:**
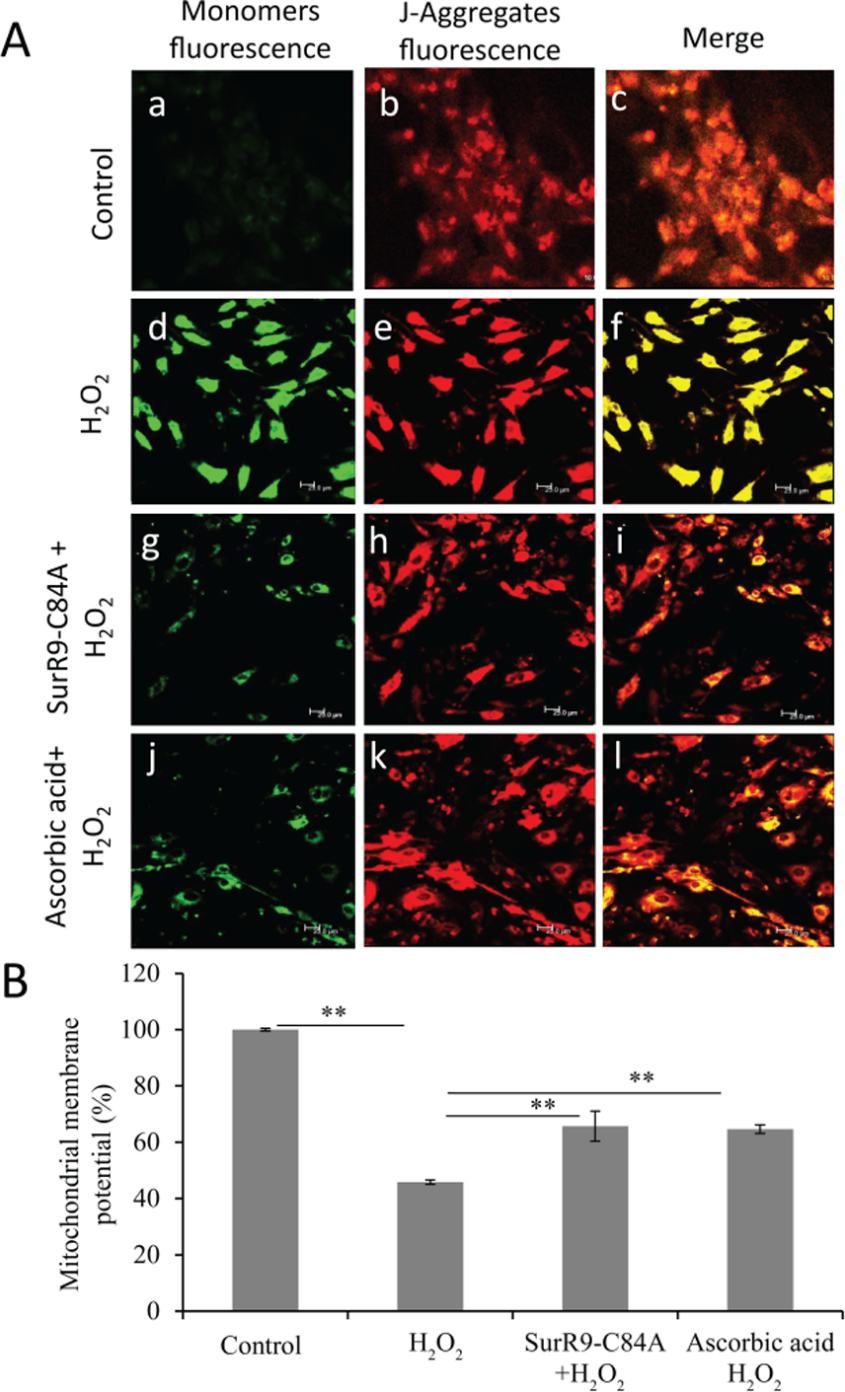
SurR9-C84A prevents mitochondrial depolarization. SK-N-SH cells were differentiated with 20 µM retinoic acid for 10 days. Differentiated media were replaced with growth media and cells were pre-treated with 75 µg/ml of SurR9-C84A or ascorbic acid for 24 hr followed by treatment with 300 µM of H_2_O_2_ for 24 hr. At the end of incubation mitochondrial membrane depolarization was qualified and quantified with MitoLight Mitochondrial kit using both techniques of (A) confocal microscopy and (B) spectrofluorometery (see material and method). Green fluorescence (detection of monomers) indicates the presence of depolarized mitochondria (apoptotic cells). Red fluorescence (J-aggregates) indicates the functional and polarized mitochondria. Values are presented as a percentage of increase in mitochondrial depolarization. Data are representative of at least three independent experiments and expressed as mean±SEM; *P<0.05, **P<0.01.

## Supporting information

S1 File Image used in panels 3G-I. (JPG)

S2 File Correct image for panels 3A-C. (JPG)
