# Supplementary figures and images for: Correction: Survivin Mutant Protects Differentiated Dopaminergic SK-N-SH Cells Against Oxidative Stress
Source: PLoS One. 2018 Mar 15;13(3):e0194587. doi: 10.1371/journal.pone.0194587 (PMC5854417; doi:10.1371/journal.pone.0194587)

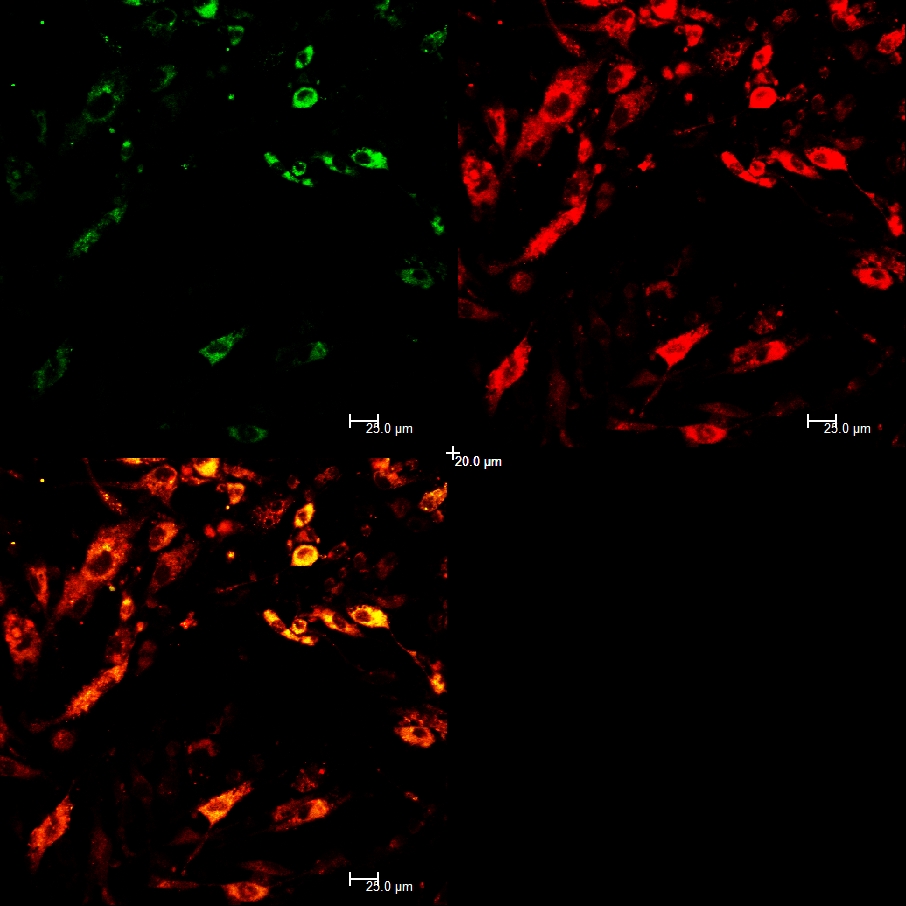

Supplement: S1 File — (JPG) [file pone.0194587.s001.jpg]

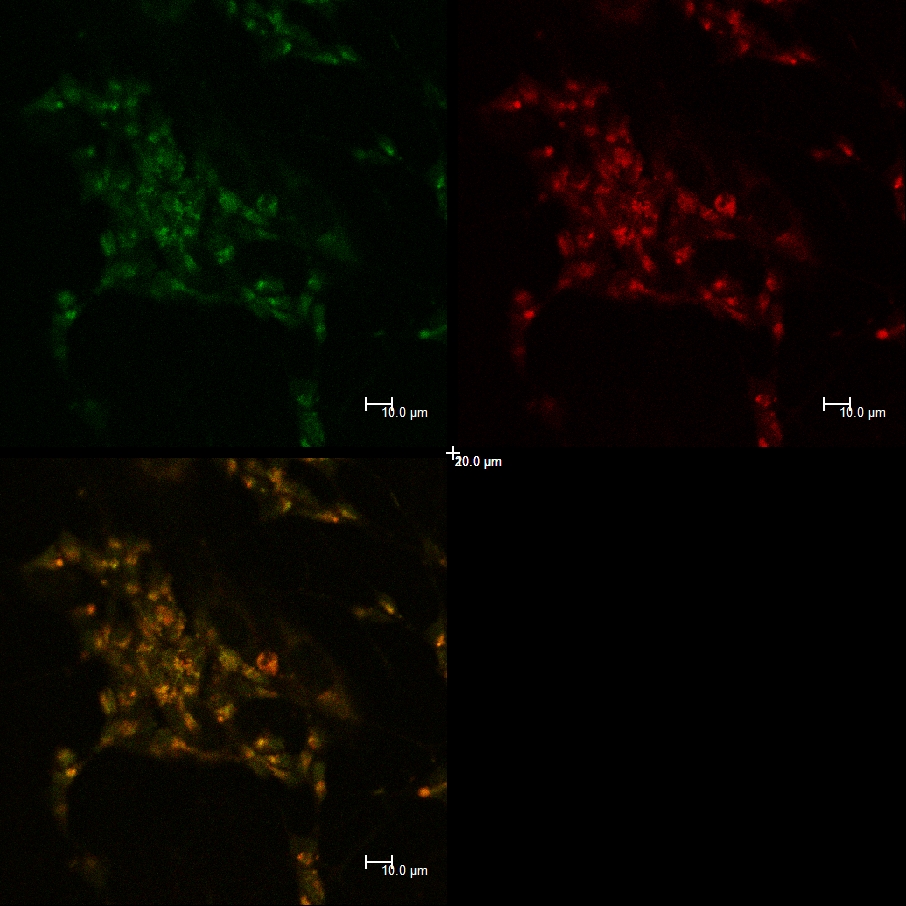

Supplement: S2 File — (JPG) [file pone.0194587.s002.jpg]
